# Evolution’s hidden architecture: a non-lipschitz theory of creation and catastrophe

**DOI:** 10.1186/s12862-025-02485-6

**Published:** 2025-12-24

**Authors:** Miguel A. Durán-Olivencia

**Affiliations:** 1 Department of Chemical Engineering, Imperial College London, London, SW7 2AZ UK; 2 Research, Vortico Tech, Málaga, 29100 Spain

**Keywords:** Macroevolution, Punctuated equilibria, Dynamical systems, Lipschitz continuity, Speciation, Extinction

## Abstract

**Background:**

Models of evolutionary dynamics have long been dominated by a paradigm of gradualism, yet the fossil record consistently points to a history defined by punctuation. This disconnect between theory and data has left major macroevolutionary events, such as punctuated equilibria, explosive radiations, and mass extinctions, without a unified first-principles explanation. We argue that this gap stems from a subtle ubiquitous assumption in theoretical models: that the underlying fitness landscapes are mathematically smooth (Lipschitz continuous).

**Results:**

We develop a theoretical framework based on relaxing this assumption, showing that non-Lipschitz dynamics are sufficient to make punctuation the default mode of evolution. We demonstrate that non-Lipschitz singularities, which arise naturally from known biological mechanisms like developmental constraints and ecological tipping points, provide a formal dynamical-systems basis for speciation as a bifurcation event and extinction as a finite-time singularity. Furthermore, we show that these dynamics are universal, appearing in contexts ranging from viral quasispecies to global biotas.

**Conclusion:**

Our framework provides a new generative engine for macroevolutionary theory. It makes specific quantitative predictions for paleontological patterns, including the decoupling of disparity and diversity during adaptive radiations, and for the genomic signatures of lineages that undergo rapid evolution. By replacing the assumption of smoothness with non-Lipschitz continuity, we offer a rigorous mathematical reconciliation between gradualist models and the punctuated nature of the fossil record.

## Background

The theoretical foundations of evolutionary biology rest on a mathematics of gradual change. The Modern Synthesis, following the tradition of Fisher and Wright [1, 2], gives us a powerful language for describing how allele frequencies shift within populations to cause gradual adaptation. Modern frameworks from quantitative genetics to adaptive dynamics are built upon this principle of continuity.

Yet, the deep-time history of life tells a different story. The fossil record reveals long periods of stasis interrupted by the geologically abrupt appearance of new species. This pattern, famously described as punctuated equilibria by Eldredge and Gould [3, 4], suggests that the tempo of evolution is fundamentally discontinuous. This observation is reinforced by larger-scale phenomena such as the “Turnover Pulse” hypothesis [5], which links pulses of speciation and extinction to climatic shifts, and “Coordinated Stasis” [6], where entire faunal assemblages persist unchanged for millions of years before rapid turnover.

Recent analyses have suggested that this punctuated mode of change is not limited to the fossil record but is a universal feature of evolving systems. Punctuational dynamics have been observed in the evolution of cancer cell lineages, viral quasispecies, and even cultural evolution [7]. Furthermore, phylogenomic studies indicate that evolution is inextricably coupled with branching events across many granularities of life [8].

How can we reconcile our smooth gradualist models with the rugged punctuated reality of macroevolution? We argue that this reconciliation does not require new evolutionary forces, but instead a revision of the mathematical properties we assume those forces possess. The vast majority of evolutionary models, whether explicitly or implicitly, assume the fitness landscape is smooth, or more formally, Lipschitz continuous. This condition implies a proportional relationship between cause and effect, where a small mutation or a slight environmental shift produces a commensurately small change in phenotype. While this is a mathematically convenient and often reasonable assumption for microevolutionary timescales, we contend it is fundamentally incorrect as a universal principle.

Here, we advance the hypothesis that the defining features of the macroevolutionary record are a necessary consequence of the failure of Lipschitz continuity in evolutionary dynamics. We present a framework where the evolutionary landscape is inherently “rugged,” a terrain characterized by cusps, cliffs, and watersheds. At these non-Lipschitz points, the standard rules of gradualism break down, and infinitesimally small perturbations can trigger massive discontinuous changes in a lineage’s evolutionary trajectory.

## Methods

### The stochastic differential equation (SDE) formalism

We model the evolutionary trajectory of a lineage as a continuous-time stochastic process. The state of the lineage at time $t$ is represented by a vector $\mathbf{x}_t$ in a high-dimensional state space $\mathcal{X}$ (e.g., a phenotype or genotype space). Its evolution is governed by an Itô Stochastic Differential Equation (SDE) of the form:

$$d\mathbf{x}_t = \mathcal{F}(\mathbf{x}_t) dt + \sigma(\mathbf{x}_t) d\mathbf{W}_t$$

This equation provides a rigorous mathematical language for describing the interplay of deterministic and stochastic forces in evolution:

- The *drift term*, $\mathcal{F}(\mathbf{x}_t) dt$, represents the deterministic component of evolutionary change over an infinitesimal time interval $dt$. It is the average direction and magnitude of change, driven primarily by natural selection and developmental constraints.
- The *diffusion term*, $\sigma(\mathbf{x}_t) d\mathbf{W}_t$, represents the stochastic component. Here, $d\mathbf{W}_t$ is a vector-valued Wiener process (or Brownian motion), representing a random uncorrelated “kick” at each time step. The matrix $\sigma(\mathbf{x}_t)$ scales the magnitude and determines the correlations of this random noise.

This SDE formalism connects directly to the foundational principles of quantitative genetics. If we interpret $\mathbf{x}_t$ as the mean phenotype of a population, then the drift vector $\mathcal{F}(\mathbf{x}_t)$ is equivalent to the force of selection on the mean phenotype, often expressed as $\mathcal{F} = \mathbf{G}\beta$, where $\mathbf{G}$ is the additive genetic variance-covariance matrix and $\beta$ is the selection gradient vector [9]. The diffusion term captures genetic drift and the input of new variation from mutation.

### Lipschitz vs. Hölder continuity

The central hypothesis of this work concerns the mathematical properties of the drift operator $\mathcal{F}$. The standard implicit assumption in many gradualistic models is that $\mathcal{F}$ is *globally Lipschitz continuous*.

#### Definition

(Lipschitz Continuity) A function $\mathcal{F}: \mathbb{R}^n \to \mathbb{R}^n$ is globally Lipschitz continuous if there exists a real constant $K \ge 0$ such that for all $\mathbf{x}, \mathbf{y} \in \mathbb{R}^n$:

$$||\mathcal{F}(\mathbf{x}) - \mathcal{F}(\mathbf{y})|| \le K||\mathbf{x}-\mathbf{y}||$$

Intuitively, this condition bounds the rate of change of the function. It states that the “steepness” of the evolutionary landscape is globally bounded; there are no points where the slope is infinite. We propose that this assumption is biologically unrealistic. Instead, we hypothesize that $\mathcal{F}$ is better characterized by the weaker condition of *Hölder continuity*.

#### Definition

(Hölder Continuity). A function $\mathcal{F}$ is Hölder continuous with exponent $\alpha \in (0, 1]$ if there exists a constant $K \ge 0$ such that for all $\mathbf{x}, \mathbf{y} \in \mathbb{R}^n$:

$$||\mathcal{F}(\mathbf{x}) - \mathcal{F}(\mathbf{y})|| \le K||\mathbf{x}-\mathbf{y}||^\alpha$$

For $\alpha=1$, this reduces to the Lipschitz condition. However, for $\alpha < 1$, the condition is significantly weaker. It allows for points where the derivative of $\mathcal{F}$ can be unbounded. A classic example is the function $f(x) = \sqrt{|x|}$, which is Hölder continuous with $\alpha = 1/2$ at $x=0$, but its derivative diverges to infinity at that point. Such a point corresponds to a “cusp” on the potential landscape.

### Simulation methods and dynamical regimes

**Fig. 1:**
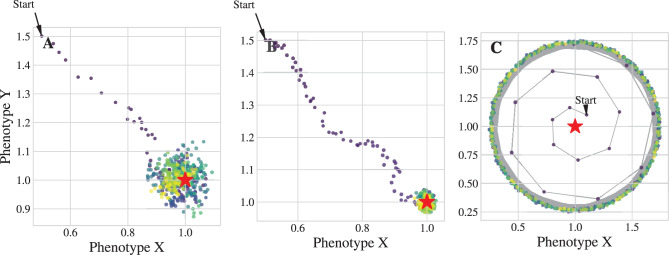
The shape of evolution. Trajectories of a lineage in phenotype space, colored by time. (**a**) On a smooth (Lipschitz) landscape, the lineage gradually converges to a stable niche, modeling stasis. (**b**) On a landscape with a sharp, non-Lipschitz cusp, the lineage can be catastrophically ejected, modeling extinction. (**c**) A different non-Lipschitz landscape can create stable orbits (limit cycles), modeling dynamic processes like co-evolutionary arms races

**Fig. 2:**
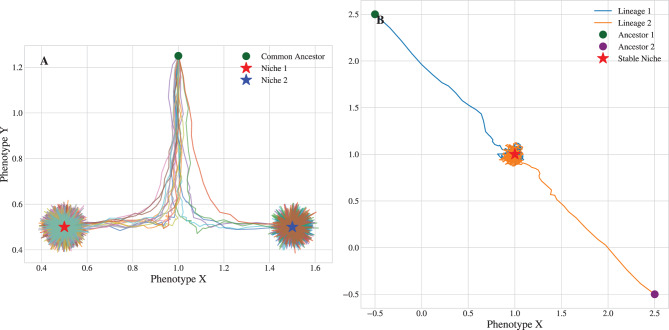
Contingency and convergence on the same landscape. (**a**) Speciation from a common ancestor. When placed on a non-Lipschitz “watershed” between two niches, tiny random variations are amplified, causing an ancestral population to split into two distinct evolutionary fates. (**b**) Convergent evolution. When starting far from the watershed, lineages with vastly different ancestors are deterministically drawn to the same stable niche, demonstrating the landscape’s power to produce predictable outcomes

The simulations were generated by numerically integrating a discrete-time stochastic map of the general form $\mathbf{x}_{k+1} = \mathbf{x}_k + \Delta \mathbf{x}_k$, which is the Euler-Maruyama approximation of the continuous-time SDE. The state vector $\mathbf{x}_k$ is two-dimensional. The update step is given by:

$$\Delta \mathbf{x}_k = \mathcal{F}(\mathbf{x}_k)\Delta t + \sqrt{2D \Delta t} \cdot \mathbf{W}_k$$

where $\mathcal{F}$ is the deterministic drift operator, $\Delta t$ is the time step (set to 0.01), $D$ is the diffusion constant (set to produce a standard deviation of 0.1 per step), and $\mathbf{W}_k$ is a vector of independent standard normal random variables. The specific deterministic drift operators $\mathcal{F}$ used in this study are canonical examples of different classes of dynamical behavior:

- **Lipschitz convergence** (Fig. 1a):

A linear restoring force, $\mathcal{F}(\mathbf{x}) = \eta(\mathbf{c}-\mathbf{x})$, derived from a quadratic potential $U(\mathbf{x}) \propto ||\mathbf{x}-\mathbf{c}||^2$.
- **Non-Lipschitz singularity** (Fig. 1b): A force derived from a potential with a fractional exponent, $U(\mathbf{x}) \propto ||\mathbf{x}-\mathbf{c}||^\alpha$ with $1 < \alpha < 2$. This creates a Hölder-continuous (but not Lipschitz) force field.
- **Non-Lipschitz limit cycle** (Fig. 1c, 3a): A non-gradient system defined in polar coordinates $(r, \theta)$ that combines a non-linear radial map pushing the state toward a circle of fixed radius with a state-dependent rotation.
- **Non-Lipschitz branching** (Fig. 2a, 2b, 3b):

**Fig. 3:**
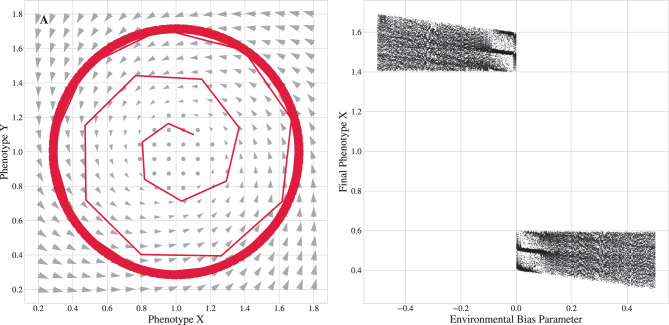
The mathematical engine of evolutionary change. (**a**) A phase portrait of the limit cycle dynamic. The arrows represent the vector field of the evolution operator, $\mathcal{F}$, showing how the deterministic “winds of selection” create a stable evolutionary vortex. (**b**) A bifurcation diagram illustrating speciation. As an environmental parameter is varied (horizontal axis), the system crosses a tipping point (parameter = 0.0). Final phenotype distributions from simulations show how a single stable lineage splits into two distinct stable descendant lineages. This illustrates the mathematical event of a supercritical pitchfork bifurcation

A force derived from a double-well potential, $U(x, y) = (x^2 - a^2)^2 + by^2$, which creates two stable minima separated by an unstable saddle point.

## Results

### A dynamical systems perspective for punctuated evolution

The distinction between Lipschitz and Hölder continuity described in the Methods has profound consequences for evolutionary trajectories.

#### Continuity and stasis

The celebrated **Picard–Lindelöf theorem** states that if the drift term $\mathcal{F}$ is Lipschitz continuous, then for any given initial condition $\mathbf{x}_0$, there exists a *unique* solution trajectory $\mathbf{x}_t$. This mathematical guarantee of uniqueness is the formal basis for a deterministic, predictable evolutionary path. Consequently, when the evolution operator $\mathcal{F}$ is Lipschitz continuous, the system is highly predictable. A lineage will follow a unique path to the nearest fitness peak and remain there, providing a clear model of evolutionary stasis (Fig. 1a).

#### Singularities and extinction

However, when the Lipschitz condition on $\mathcal{F}$ fails (as it does at the “cusps” characteristic of a Hölder-continuous operator) this fundamental guarantee of uniqueness breaks down [10]. One consequence is that dynamics can lead to solutions that “explode” to infinity in a finite amount of time. In our model, a singularity in the fitness landscape can violently repel a lineage, causing it to escape to an unviable region of phenotype space in a finite amount of time, a formal model for extinction (Fig. 1b). Other non-Lipschitz forms can trap lineages in stable orbits (limit cycles), which model the dynamic equilibria found in predator-prey coevolution (Fig. 1c).

#### Non-uniqueness and speciation

The second consequence of Lipschitz failure is non-uniqueness. At a non-Lipschitz point, the SDE can admit multiple distinct future solutions from the same initial condition. Critically, our framework provides a mechanism for the origin of new lineages based on this property. A watershed ridge between two fitness peaks is a non-Lipschitz region where the uniqueness of evolutionary trajectories is lost. On this ridge, a population is inherently unstable. Stochastic noise becomes amplified, deterministically splitting the population into two distinct descendant lineages that diverge toward separate peaks (Fig. 2a). This process provides a rigorous mechanism for speciation that does not require geographic isolation.

#### Bifurcation as a phase transition

The connection between macroevolution and the physics of complex systems can be made more explicit by analyzing this speciation event as a critical phenomenon, or phase transition [11, 12]. We consider the double-well potential model with an added environmental control parameter $h$ that introduces an asymmetry:

$$U(x,y) = (x^2 - a^2)^2 + by^2 - hx$$

The event can be formally characterized as a bifurcation (Fig. 3b). As the control parameter changes, representing a slow environmental or geological shift, the system crosses a critical tipping point ($h=0$) causing one stable phenotype to split into two via a supercritical pitchfork bifurcation. This bifurcation is formally equivalent to a *second-order phase transition* in physics, such as the spontaneous magnetization of a ferromagnet below the Curie temperature.

### The biological and physical basis of a rugged landscape

This rugged landscape is not a mathematical contrivance but an outcome of biological complexity. We identify several primary drivers of non-Lipschitz dynamics:

- **Abiotic control parameters:** The external environment can impose non-Lipschitz dynamics directly. Ecosystems can undergo sudden, critical transitions, such as the collapse of a forest ecosystem due to gradual climate change [13]. Geological changes or climate shifts act as control parameters that alter the landscape’s topology, driving “turnover pulses” [5].

- **The genotype-phenotype map as a rugged landscape:** Our mathematical landscape finds its physical origin in the complex multi-layered process of development that translates genetic information into a functional organism. The Genotype-Phenotype (G-P) map is the central object that shapes the evolutionary operator acting on the genome. A complex and non-linear G-P map can induce a highly rugged non-Lipschitz effective landscape on the underlying genotype space. This is grounded in well-established concepts from evolutionary developmental biology (Evo-Devo):

- *Canalization:* Described by C.H. Waddington [14], canalization is the process by which developmental pathways are buffered against perturbations, ensuring a consistent phenotypic outcome. In our landscape model, canalized phenotypes correspond to deep wide valleys, the Lipschitz regions of the state space that produce stasis.

- *Modularity:* Organisms are composed of quasi-independent modules that can evolve semi-independently [15, 16]. The interfaces and connections between these modules can be sources of developmental constraint and innovation, forming the ridges and watersheds of the landscape.

- **Molecular mechanisms of discontinuity:** The “wrinkles” and “cliffs” on the G-P landscape are generated by specific molecular processes that can induce large, discontinuous phenotypic changes from small genetic alterations.

- *Transposable elements (TEs):* TEs are a powerful engine of genomic and phenotypic novelty [17]. Unlike point mutations, the mobilization of a TE is a discrete, large-scale event that can disrupt genes, alter regulation, or mediate genomic rearrangements, representing a potential “jump” across the G-P landscape [18].

- *Intrinsically disordered proteins (IDPs):* IDPs possess significant regions that remain unfolded and highly flexible [19]. This structural ductility allows them to bind to a wide range of molecular partners. Because function is not tied to a rigid structure, IDP sequences can evolve rapidly, leading to the abrupt emergence of novel cellular functions [20].

- **Ecological feedback (niche construction):** The evolutionary landscape is not static. It is dynamically shaped by both external environmental changes and the actions of the organisms themselves. *Niche Construction Theory (NCT)* posits that organisms are active agents that modify their environments [21, 22]. This introduces a crucial feedback loop into the evolutionary dynamic: the landscape shapes the organism, and the organism, in turn, reshapes the landscape. This feedback can either smooth out landscapes or sharpen them, potentially creating the very tipping points that drive non-Lipschitz events.

## Discussion

### Relation to other frameworks

This non-Lipschitz framework synthesizes several concepts in evolutionary theory. It provides a concrete physical mechanism for the abstract state-changes of *Catastrophe Theory* [23] and offers a local dynamical mechanism for the emergent statistical patterns of *Self-Organized Criticality (SOC)* [24]. It also formalizes the “frustrated” landscapes described by Niklas [25] and the interplay between developmental and ecological drivers discussed by Marshall [26]. Below we provide a more detailed comparison with these existing theories:

- **Self-organized criticality (SOC)**: The theory of *Self-Organized Criticality*, particularly the Bak-Sneppen model of evolution, proposes that complex ecosystems naturally evolve toward a critical state, poised on the edge of instability [24, 27]. In this state, a small perturbation can trigger an “avalanche” of co-extinctions of all possible sizes, following a power-law distribution. This model successfully reproduces the statistical pattern of punctuated equilibrium [28]. SOC is a powerful phenomenological model that describes the emergent statistical properties of a complex system. Our framework provides a candidate *mechanism* for the individual events that constitute the SOC dynamic. Each “avalanche” can be viewed as the consequence of one or more lineages crossing a non-Lipschitz critical point on the landscape.

- **Catastrophe theory**: Developed by René Thom, *Catastrophe Theory* is a branch of bifurcation theory that classifies how the stable equilibria of a dynamical system can change abruptly as control parameters are varied smoothly [23]. It provides a rigorous mathematical language for “tipping points” [29]. Our framework is deeply related to the spirit of Catastrophe Theory but is distinct and more general. Catastrophe Theory is a *topological classification* of the stable bifurcations of gradient systems in low-dimensional spaces. Our framework, in contrast, focuses on a local mathematical property of the evolution operator itself, its failure to be Lipschitz continuous, which is the *cause* of such bifurcations. It is also more general, applying to non-gradient systems (like co-evolutionary arms races) and high-dimensional spaces.

- **Computational phylogenetics**: Our results parallel recent findings using Bayesian phylogenetic tools (e.g., BEAST). Just as the “molecular clock” is a built-in bias towards gradualism in phylogenetics, Lipschitz continuity is a bias in dynamical modeling. When researchers relax the strict molecular clock assumption in tools like BEAST, punctuational patterns naturally emerge in the resulting trees [8]. Similarly, our work shows that relaxing the Lipschitz assumption naturally yields punctuational dynamics in phenotype space.

- **Reforming the adaptive landscape**: Recent historical analyses have argued that Wright’s original adaptive landscape metaphor is “deeply flawed” and requires reformulation within the Extended Evolutionary Synthesis [30]. Our work directly addresses these historical critiques by demonstrating that the assumption of smoothness (Lipschitz continuity) is the specific mathematical constraint that must be relaxed to make the landscape model empirically fecund and capable of explaining macroevolutionary dynamics.

### A comprehensive program for empirical validation

The primary value of our framework lies in its ability to generate specific quantitative predictions that distinguish it from gradualist models (see Table 1).

#### Hypothesis 1 (paleontology) - disparity precedes diversity

Our model predicts that in the wake of a mass extinction, the exploration of vacant ecospace is driven by non-Lipschitz repulsion from unviable regions. This leads to a rapid increase in morphological novelty that precedes the slower accumulation of species. **Test:** We predict that *disparity should increase significantly faster than diversity during recovery phases*. This can be tested by comparing disparity-diversity scaling exponents. **Refined test and complexity:** While the empirical relationship between disparity and diversity is known to be complex [31], our theory provides a generative cause for this decoupling specifically in the recovery phase [32]. A formal statistical test would involve compiling time-series data for both taxonomic diversity and morphological disparity for clades that radiated after major mass extinctions [33]. The non-Lipschitz model predicts that a log-log plot of disparity versus diversity during the post-extinction recovery phase will show a significantly higher scaling exponent than the same plot for clades diversifying during background times.

#### Hypothesis 2 (genomics) - the fractal geometry of the G-P map

If the landscape is non-Lipschitz, the standard Gaussian assumptions of quantitative genetics must fail. **Prediction:** If the internal developmental source of discontinuity is a primary driver of macroevolution, the distribution of fitness effects (DFE) of new mutations should be *heavy-tailed*, reflecting the fact that while most mutations have small effects, there is a non-negligible probability of rare mutations with very large phenotypic consequences [34]. The expected distribution would be a power-law or a related heavy-tailed form, such as a Lévy-stable distribution [35–37]. This reflects a landscape of many small steps punctuated by rare large leaps. Such distributions should be detectable in Deep Mutational Scanning (DMS) datasets, contrasting with the exponential tails of smooth landscape models. **Evidence and methods:** This prediction can be tested directly using data from high-throughput mutagenesis experiments like Deep Mutational Scanning (DMS) [38]. A meta-analysis of existing DMS datasets should show that the resulting DFEs are statistically better fit by heavy-tailed distributions than by distributions with exponential tails (e.g., Gaussian, exponential) [39, 40].

#### Hypothesis 3 (comparative genomics) - signatures of radiations

If mechanisms like TEs and IDPs drive macroevolutionary leaps, their activity should peak during these events. **Prediction:** Therefore, *clades characterized by exceptionally rapid adaptive radiations will show evidence in their genomes of a higher ancestral rate of TE activity and/or a significant expansion of IDP-coding gene families* compared to their non-radiating sister taxa. **Test:** We predict that phylogenetic comparative methods will show a significant increase in the rate of TE accumulation and/or IDP gene family expansion on the ancestral branches subtending major adaptive radiations. **Case study:** The explosive adaptive radiations of cichlid fishes in the Great Lakes of Africa provide an ideal system to test this prediction. Genomic studies have already revealed that their evolution is associated with an excess of gene duplications, accelerated coding sequence evolution, and, critically, expression divergence linked to TE insertions [41–43]. A formal phylogenetic comparative analysis should identify a significantly accelerated rate of TE accumulation and/or IDP gene family expansion on the specific branches of the phylogeny that subtend the major adaptive radiations.

**Table 1:** Overview of falsifiable predictions distinguishing non-Lipschitz dynamics from gradualist models. We contrast the core predictions of our framework with standard expectations, identifying specific data sources and statistical signatures for validation

Hypothesis	Core prediction	Data source	Statistical signature
H1: Disparity precedes diversity	Morphological disparity increases faster than taxonomic diversity after mass extinctions.	Fossil databases	Higher scaling exponent in log-log disparity vs. diversity plots.
H2: Fractal G-P map	The distribution of phenotypic effects of new mutations is heavy-tailed.	Deep mutational scanning (DMS) datasets	DFE fits power-law/Lévy distributions better than Gaussian/exponential.
H3: Genomic drivers of radiation	Radiating clades are ancestrally enriched for molecular discontinuity drivers.	Comparative genomics of adaptive radiations	Elevated rates of TE accumulation and IDP expansion.

### Limitations

We acknowledge that the “adaptive landscape” is a metaphor that simplifies the high-dimensional reality of biological evolution. However, the dynamical systems properties we describe, specifically the loss of uniqueness and stability at non-Lipschitz points, are robust features of differential equations that describe state changes, whether those states are allele frequencies or morphological vectors.

## Conclusions

By replacing the pervasive assumption of smoothness with the more biologically realistic condition of non-Lipschitz continuity, we have developed a framework that unifies the punctuated and gradual modes of evolution. This theory provides a new generative engine for macroevolutionary modeling and offers a clear quantitative program for empirical testing.

## Data Availability

No datasets were generated or analysed during the current study.

## Abbreviations

G-P: Genotype-Phenotype
TE: Transposable Element
IDP: Intrinsically Disordered Protein
SOC: Self-Organized Criticality
DFE: Distribution of Fitness Effects
DMS: Deep Mutational Scanning
SDE: Stochastic Differential Equation
